# Finerenone and other future therapeutic options for Alport syndrome

**DOI:** 10.1007/s44162-023-00022-x

**Published:** 2023-11-08

**Authors:** Helen Pearce, Holly Mabillard

**Affiliations:** 1 Renal Services, The Newcastle upon Tyne Hospitals NHS Foundation Trust, Newcastle upon Tyne, NE7 7DN UK; 2 Translational and Clinical Research Institute, Faculty of Medical Sciences, Newcastle University, Central Parkway, Newcastle upon Tyne, NE1 3BZ UK; 3 NIHR Newcastle Biomedical Research Centre, Newcastle University, NE4 5PL UK

**Keywords:** Chronic kidney disease, Alport syndrome, Renin-angiotensin system, Mineralocorticoid receptor antagonists, Kidney fibrosis

## Abstract

Alport syndrome is a rare genetic disease that results in disordered basement membrane type IV collagen resulting in haematuria, proteinuria and often development of renal fibrosis leading to progressive kidney disease. The therapeutic blockage of the renin-angiotensin-aldosterone system, which slows the progression to kidney failure, is supported by strong evidence. Recent clinical trials using sodium-glucose co-transporter-2 (SGLT2) inhibitors and mineralocorticoid receptor antagonists (MRA) in patients with chronic kidney disease have changed the therapeutic landscape. Patients with Alport syndrome and progressive kidney disease may benefit from treatment with MRAs because research has shown that these drugs are nephroprotective through a variety of mechanisms, including by preventing fibrosis. Ongoing clinical trials show great promise in order to help establish the long-term safety and efficacy of Finerenone, a MRA. This review discusses the evidence for the use of MRAs as a potential treatment in Alport syndrome that may slow the progression of chronic kidney disease and prevent patients reaching kidney failure.

## Introduction

In the last few years, the pharmacological management of patients with chronic kidney disease (CKD), including Alport syndrome (AS) has changed significantly [1]. Clinical trials have highlighted the beneficial effects of the use of renin–angiotensin system (RAAS) blockers on renal outcomes (angiotensin-converting–enzyme [ACE] inhibitors or angiotensin-receptor blockers [ARB]) and, more recently, sodium–glucose co-transporter 2 (SGLT2) inhibitors [2, 3]. However, despite the use of these, the need for new therapies remains. Finerenone is a selective, nonsteroidal mineralocorticoid receptor antagonist (MRA). It differs to steroidal mineralocorticoid receptor antagonists due to increased potency and selectivity for mineralocorticoid receptors, shorter half-life and lack of active metabolites [4]. In brief, its nephroprotective effects revolve around preservation of kidney structures due to its combined anti-fibrotic, anti-inflammatory and anti-oxidative effects [5]. A clinical trial demonstrated that in patients with CKD and type 2 diabetes (T2DM), treatment with Finerenone resulted in lower risks of CKD progression and cardiovascular events when compared to a placebo [1]. This poses the question whether the use of MRAs such as Finerenone may be beneficial in the management of patients at risk of renal fibrosis leading to a progressive loss of kidney function, for example those with AS, which commonly leads to kidney failure at a relatively young age.

### Alport syndrome: disease mechanism and therapeutic targets

AS is a rare, hereditary disease resulting in structural and functional abnormalities in the glomerular basement membrane (GBM) [6]. The GBM is an extracellular matrix (ECM) which acts as a semipermeable glomerular filtration membrane. The GBM acts as a selective barrier, crucial to preventing the passage of blood and protein molecules from entering the urine via the blood. The ECM is composed of a number of macromolecules, essential for its integrity. These include: type IV collagen, laminin, agrin and nidogen. Type IV collagen makes up approximately 50% of the GBMs protein mass and is therefore pivotal in its ability to function effectively [7].

Alport syndrome is characterised by variants in the *COL4A3, COLA4* and *COL4A5* genes which code for type IV collagen α3-α4-α5 chains, respectively. AS results in kidney, auditory and ocular involvement with these three organs unified by the presence of α3-α4-α5(IV) in their basement membranes. AS has three modes of inheritance: X-linked AS, autosomal and digenic [8]. Disease severity is influenced by the nature and position of the associated *COL4A* mutations. The typical histological kidney lesion observed in patients with established AS is widespread, irregular thickening and thinning of the GBM and what has been previously described as a basket weave appearance when viewed on electron microscopy [9]. These structural abnormalities and subsequent dysfunction of the GBM in patients with AS typically result in proteinuria and haematuria. As the disease process progresses chronic inflammation occurs, leading to kidney fibrosis [6, 7].

Progression to kidney failure in AS patients is variable and difficult to predict, however it is informed by genetic and environmental factors. At present there is no ‘cure’ for AS, however, there are a number of pharmacological targets which have been shown to slow progression to kidney failure. In particular, ACE inhibitors, targeting the renin-angiotensin system are currently used in the treatment of AS. ACE inhibitors have been shown to be nephroprotective due to their anti-hypertensive, anti-fibrotic and anti-proteinuric properties. Additionally, Gross et al. demonstrated that ACE inhibitors help to prevent podocyte injury, by lowering glomerular filtration pressure [10]. However, despite treatment with ACE inhibitors, AS patients with a severe phenotype continue to reach kidney failure in their early adult life. In this regard, treatment with ACE inhibitors slows but does not stop the progression to kidney failure in AS and emphasises the urgent need for the discovery of new treatment options [11].

More recently, several clinical trials have highlighted the nephroprotective effects of SGLT2 inhibitors in patients with CKD. SGLT2 inhibitors have been proven to reduce intraglomerular pressure and albuminuria, thus helping to stabilise kidney function, alongside protective anti-inflammatory and anti-oxidative properties. These cumulative effects may mean SGLT2 inhibitors have potential to be beneficial in the management of AS [12–14].

### Nephroprotective mechanisms of Finerenone

Hyperaldosteronism is observed in patients with CKD. Despite these patients being prescribed long-term ACE inhibitors/ARB therapy, increased aldosterone levels continue to be observed. High levels of aldosterone are associated with pro-inflammatory and pro-fibrotic pathways, both of which are known to contribute to CKD and atherosclerotic cardiovascular disease (CVD) [5, 15].

Aldosterone binds to mineralocorticoid receptors (MRs), which belong to the nuclear receptor superfamily. On binding, aldosterone promotes a conformational change and translocation from the cell cytoplasm to the nucleus. The MR then recruits transcriptional cofactors and induces the transcription or repression of its corresponding genes [16]. Two steroidal MR antagonists, Spironolactone and Eplerenone have been proven to be beneficial in both the management of cardiovascular disease (CVD) and improvement in proteinuria observed in patients with diabetic kidney disease. However, the use of these medications is limited due to the ongoing risk of hyperkalaemia, especially seen in patients with CKD. In light of ongoing safety concerns, nonsteroidal mineralocorticoid receptor antagonists were developed [5].

**Fig. 1:**
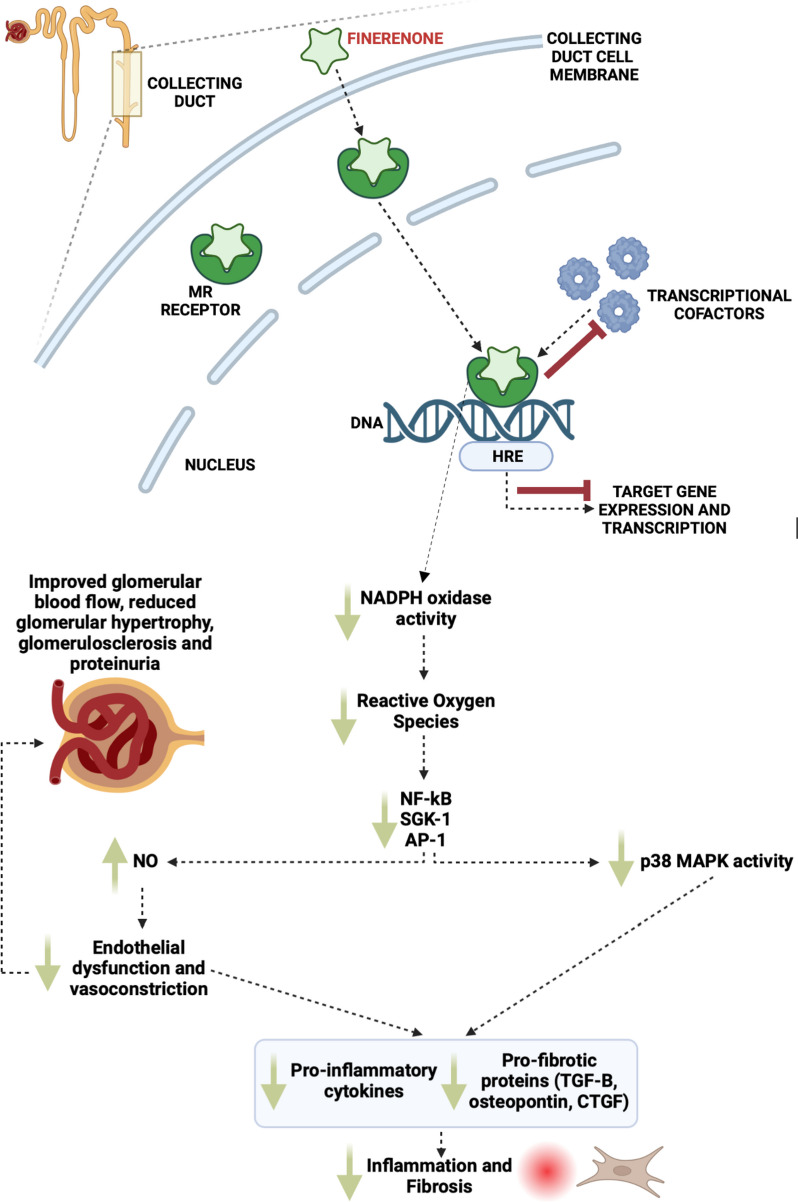
Finerenone and its mechanism of action in the kidney. The non-steroidal mineralocorticoid receptor antagonist, Finerenone, competitively binds to mineralocorticoid receptors (MR) which promotes a conformational change once translocated into the nucleus. Normally, Aldosterone overexpression in diseased states triggers the MR to bind to hormone response elements, recruit transcriptional cofactors and target the initiation of target gene transcription. This initiates subsequent expression of pro-inflammatory and profibrotic genes and their associated signalling pathways. Finerenone inhibits transcriptional cofactor recruitment to the MR in the absence of Aldosterone and it inhibits target gene transcription and expression. The anti-inflammatory and anti-fibrotic activity of Finerenone is more potent and selective than that of steroidal mineralocorticoid antagonists such as Spironolactone or Eplerenone, as is detailed in the bottom half of the figure, including on its impact on the kidney. Its beneficial effects on reducing glomerular filtration pressure, glomerular damage and ameliorated inflammation/fibrosis makes it an ideal candidate for Alport syndrome in which the predominant pathophysiology is focussed on these structures and mechanisms. Adapted from Kim et al. [4]. AP-1; activator protein-1; CTGF, connective tissue growth factor; HRE, Hormone Response Elements; MAPK, mitogen-activated protein kinase; MR, mineralocorticoid receptor; NADPH oxidase, nicotinamide adenine dinucleotide phosphate-oxidase; NF-kB, nuclear factor-kB; NO, nitric oxide; SGK-1, serum and glucocorticoid-induced protein kinase-1; TGF-B, transforming growth factor-B.

Finerenone is a selective, nonsteroidal mineralocorticoid receptor antagonist (MRA), which on binding to MRs, results in a conformational change, thus leading to aldosterone inhibition. Finerenone also suppresses the recruitment of transcriptional cofactors and decreases the accumulation and turnover of MRs in the nucleus. Studies suggest that Finerenone elicits its nephroprotective mechanisms through its anti-fibrotic, anti-inflammatory and anti-oxidative effects, thus aiding in the preservation of kidney structures. In comparison to steroidal MRAs, Finerenone does not produce active metabolites and has a short half-life of 2-3 hours. Additionally, Finerenone has been shown to be more evenly distributed between the kidney and the heart [4]. Together, these reduce the risk of kidney accumulation and result in less risk of hyperkalaemia [5]. See Fig. 1 for an overview of Finerenone’s protective mechanism of action.

In contrast to Finerenone, SGLT2 inhibitors target a different mechanism within the kidneys. Inhibition of SGLT2, blocks glucose absorption in the proximal convoluted tubule. This results in glycosuria, which in turn reduces blood glucose levels. Additionally, studies suggest that SGLT2 inhibitors work independently of glycaemic control by reducing albuminuria and glomerular hyperfiltration [13].

### The use of Finerenone in renal disease- the evidence

The FIDELIO-DKD trial (clinical trial number NCT02540993) and the FIGARO-DKD trial (clinical trial number NCT02545049), funded by Bayer, together form the largest cardiorenal outcomes programme for patients with both CKD and T2DM. These are both phase three, randomised, double-blind, placebo-controlled, multicentre trials in adult participants with both CKD and T2DM. They differ however in that the FIDELIO-DKD trial was designed to detect a treatment effect of Finerenone on kidney failure endpoints, whereas the FIGARO-DKD aimed to detect the effect of Finerenone on cardiovascular disease as a composite primary endpoint. The FIDELITY pooled analysis then went on to combine results from FIDELIO-DKD and FIGARO-DKD trials to help establish the overall effects of Finerenone on both CKD progression and CVD in patients with both CKD and T2DM [1, 17, 18].

In FIDELIO-DKD patients satisfying the following inclusion criteria were eligible to join the trial: ≥ 18 years, a urinary albumin-to-creatinine (UACR) ratio of 30 to less than 300 (mg/g), an estimated glomerular filtration rate (eGFR) of 25 to less than 60 ml per minute per 1.73 m^2^ of body-surface area, and diabetic retinopathy, or they had a urinary ACR of 300-5000 and an eGFR of 25 to less than 75 ml per minute per 1.73 m^2^ and serum potassium $\le$ 4.8 mmol/L. Prior to commencement of the trial, all eligible patients received RAAS blockade at the manufacturers highest recommended dose. FIDELIO-DKD primary outcome was a composite of kidney failure, sustained decrease of at least 40% in the eGFR from baseline, or death from kidney causes. Kidney failure was defined as an eGFR of less than 15 ml per minute per 1.73 m^2^ or kidney failure requiring renal replacement therapy. An important secondary outcome was death from cardiovascular causes, non-fatal stroke or myocardial infarction, or hospitalisation for heart failure [1].

FIDELIO-DKD demonstrated that in 5734 patients randomised and followed over a median 2.6 years, Finerenone was associated with a lower incidence of sustained decline in eGFR of ≥40%, kidney failure, or kidney death (504 patients, 17.8%) compared with the placebo group ((600 patients, 21.1%) hazard ratio 0.82; 95% confidence interval [CI], 0.73 to 0.93; *P* = 0.001)). The positive effects of Finerenone on the primary outcome were seen consistently across several pre-specified subgroups. Additionally, in the Finerenone group, the secondary outcome occurred in 367 patients (13.0%) and 420 patients (14.8%) in the placebo group (hazard ratio, 0.86; 95% CI, 0.75 to 0.99; *P*=0.03). Overall, the FIDELIO-DKD trial concluded that Finerenone was effective in lowering the risk of CKD progression and incidence of cardiovascular events when compared to those not receiving Finerenone.

For the FIGARO-DKD trial patients were only eligible if they had T2DM and CKD (defined as UACR 30–<300 mg/g and eGFR 25–90 mL/min/1.73 m 2, or UACR 300–5000 mg/g and eGFR >_60 mL/min/1.73 m^2^) The study primary outcome was a composite of death from cardiovascular causes, non-fatal stroke or myocardial infarction, or hospitalization for heart failure. Secondary outcome included the first occurrence of kidney failure, a sustained decrease from baseline of at least 40% in the eGFR for a minimum of four weeks, or death from kidney causes. Results from the trial demonstrated that in 7352 patients who were randomised and followed up over a median of 3.4 years, the incidence of the primary composite outcome was significantly lower in the Finerenone group in comparison to the placebo group (458 of 3686 patients [12.4%] vs. 519 of 3666 patients [14.2%]; hazard ratio, 0.87; 95% confidence interval [CI], 0.76 to 0.98; *P*=0.03)). There was no reported significant difference in the main secondary outcome in the Finerenone group (350 patients [9.5%]) compared to the placebo group (395 [10.8%]) (hazard ratio, 0.87; 95% CI, 0.76 to 1.01) [18].

The FIDELITY pooled analysis went on to combine results from FIDELIO-DKD and FIGARO-DKD trials comprising 13,026 patients with a broad spectrum of CKD and T2DM, all treated of whom were receiving the manufacturers’ maximum stated dose of an ACE inhibitor or ARB therapy. Pooled results showed that the relative risk reduction was 23% for the composite kidney outcome and 14% for the composite cardiovascular outcome. In FIDELITY, hyperkalaemia was more frequently associated with the Finerenone group vs. the placebo group. However, the reported number of adverse effects related to hyperkalaemia was low. Permanent discontinuation of treatment in the Finerenone group was seen in 1.7% of patients vs. 0.6% of patients in the placebo group over a median follow-up period of 3 years [18].

Additionally, two clinical trials have been initiated looking at the effect of Finerenone on patients with CKD, in the absence of T2DM. The FIND-CKD trial (clinical trial number NCT05047263) started in September 2021 and has an estimated primary completion date of January 2026. It is a randomised, double-blind, placebo-controlled, multicentre phase three trial in adults with CKD. Patients included had a UACR ≥200 but ≤3500 mg/g. Finerenone is being tested in patients with CKD with moderately to severely increased albuminuria of non-metabolic origin (patients without diabetes). The FIONA OLE trial (clinical trial number NCT05457283) started in November 2022, with an estimated completion date of September 2028. The aim of this trial is to establish the long-term safety and efficacy of Finerenone given in addition to either an ACE inhibitor or ARB with the aim to reduce the progression of CKD in patients under the age of 18 years.

### Safety of Finerenone

In recent years, several clinical researchers have focused on the efficacy and safety of Finerenone. Data from a meta-analysis reported that aside from concerns regarding the association between Finerenone and an increased risk of hyperkalaemia, there was no reported significant difference in the incidence of adverse events between those receiving Finerenone compared to those receiving the placebo [19].

The use of MRAs has been associated with an increased risk of hyperkalaemia, especially in those with CKD who are prone to high potassium levels. Although Finerenone is less likely to be associated with hyperkalaemia, when compared to steroidal MRAs, safety concerns remain. Pitt et al. [20] investigated the incidence of hyperkalaemia in patients with heart failure and CKD who were administered Spironolactone (steroidal MRA) or Finerenone; the rate of hyperkalaemia (12.7% vs. 5.3%, *P*=0.048) in the Spironolactone group was significantly higher than those in the Finerenone group. Filippatos et al. demonstrated that there was a far greater increase in serum potassium levels from their baseline in patients assigned to the receiving Eplerenone compared to those receiving Finerenone [21].

The FIDELIO-DKD trial (clinical trial number NCT02540993) reported the incidence of adverse effects to be similar between the placebo and Finerenone group for the duration of the clinical trial [1]. Although no fatal hyperkalaemic episodes were recorded in either of the groups, in the first four months of treatment, patients assigned to receive Finerenone had a higher mean serum potassium level, which was reported to have remained stable after the initial four-month period. Overall, the incidence of serum potassium levels > 5.5 mmol/l and > 6.0 mmol/l were 21.7% and 4.5%, respectively, in the Finerenone group and 9.8% and 1.4%, respectively, in the placebo group [1].

Aside from the risk of hyperkalaemia, some mild, treatment emergent adverse effects have been associated with Finerenone, these include: nasopharyngitis, constipation, bronchitis and a reduction in systolic blood pressure [20]. Data from 6 studies showed that the incidence of total adverse events (TAE) in the Finerenone group versus the placebo was not statistically different [1, 19–23].

### Future therapeutic options in Alport syndrome

ACE inhibitors and ARBs form the current mainstay treatment for CKD in AS. However, in the past few years’ interest in the development of new treatments has increased significantly. There are now several well-established patient-founded organisations, specific to AS, to help facilitate, develop and aid in the progression clinical trials, hoped to lead to the discovery of new and novel treatments for AS [24].

### SGLT2 Inhibitors

SGLT2 inhibitors block glucose absorption in the proximal convoluted tubule, thus promoting glycosuria and reduction in blood glucose. Renal outcomes in both non-diabetic and diabetic patients taking SGLT2 inhibitors have been to improve, thus indicating the benefit of SGLT2 inhibitors extends beyond use in glycaemic control [24]. SGLT2 inhibitors have several nephroprotective properties, including reduction in glomerular filtration pressures and albuminuria, helping to stabilise kidney function [12, 13]. In addition, treatment with SGLT2 inhibitor Empagliflozin reduces lipid droplet accumulation and apoptosis in AS podocytes, thus reducing renal lipotoxicity [25].

DAPA-CKD (clinical trial number NCT03036150) showed that among patients with CKD, regardless of their diabetic status, Dapagliflozin reduced the risk of a composite of a sustained decline in the estimated GFR of at least 50%, kidney failure, or death from a kidney or cardiovascular cause [26]. Clinical trials specific to SGLT2 inhibitors provide a growing body of evidence for the potential benefits of their use in AS.

### Bardoxolone methyl

Bardoxolone methyl is a semisynthetic triterpenoid. It elicits its effects through activation of Nrf2 (nuclear factor erythroid-derived 2-related factor 2) and inhibition of NF-κB (nuclear factor kappa-light-chain-enhancer of activated B-cells) thus modulating the expression of multiple genes involved in oxidative stress, inflammation and cellular energy metabolism. In the BEAM trial which began in 2011 (NCT00811889), Bardoxolone methyl was shown to improve eGFR in patients with CKD and T2DM over a 52-week period [27]. Following on from the BEAM trial, the BEACON trial (clinical trial number NCT01351675) emerged. This trial concluded that among patients with stage 4 CKD and T2DM, Bardoxolone methyl failed to reduce the risk of kidney failure or death from cardiovascular causes. Results demonstrated a higher rate of cardiovascular events with Bardoxolone methyl than with placebo leading to termination of the BEACON trial [28].

In 2017, the CARDINAL trial (NCT03019185) emerged, involving AS patients. Disappointedly, this phase three, international, multicentre, double-blind, placebo-controlled, randomised registrational trial failed to demonstrate nephroprotective effects of Bardoxolone, instead, Bardoxolone treated patients were found to have increased liver enzymes consistent with chronic liver toxicity. And although Bardoxolone methyl was associated with an increase in eGFR it also led to a concomitant increase in geometric mean UACR, thus presumably resulting in increased glomerular pressure [29]. This raised concerns regarding the long-term safety of Bardoxolone methyl in AS patients.

### Sparsentan

Endothelin 1 is a vasoactive peptide with pleiotropic effects in renal pathophysiology and is an important factor in the progression of AS. Endothelin-1 is an agonist for endothelin type A receptor and endothelin type B receptor. Activation of ET_A_R results in vasoconstriction, inflammation and fibrosis [30].

Sparsentan is a dual endothelin type A receptor/angiotensin II type 1 receptor inhibitor with high selectivity for the ETA receptor and angiotensin II subtype 1 receptor (AT1 receptor). Sparsentan has been shown to reduce proteinuria, increased lifespan, and improved auditory abnormalities in mice models with AS [31]. The DUPLEX study (clinical trial number NCT03493685) aimed to assess the long-term nephroprotective potential of treatment with Sparsentan as compared to ARBs in patients with focal segmental glomerulosclerosis (FSGS) [32]. With an estimated study completion date of February 2026, results may have implications for patients with AS, as FSGS falls under the spectrum of AS [8]. Additionally, the PROTECT clinical trial (clinical trial number NCT03762850), a phase 3 clinical trial, investigated the potential use of Sparsentan in adults with IgA nephropathy. It concluded that once-daily treatment with Sparsentan produced meaningful reduction in proteinuria compared with Irbesartan in adults with IgA nephropathy [33].

The EPPIK study (clinical trial number NCT05003986) is currently underway and has recruited paediatric patients with glomerular disease and proteinuria, including those with AS. The study aims to evaluate the safety, efficacy and tolerability of the use of Sparsentan and assess changes in proteinuria over the 108-week period in patients with glomerular disease. Once again, results from this trial may provide promising evidence to support the use of Sparsentan in patients with AS.

### Hydroxychloroquine

Hydroxychloroquine is an immunomodulatory drug used in the management of malaria and a number of rheumatological diseases. It elicits its effects on the immune system through inhibition of toll-like receptor signalling, thus suppressing cytokine production. Studies suggest that hydroxychloroquine reduces the production of cytokines, including TNF-α, IL-6, IFN-α. It is possible that the inhibition of TNF-α, IFN-α and IL-6 expression alleviates the inflammation response resulting from mesangial cell activation. By inhibiting this specific part of the immune response, hydroxychloroquine may help to reduce podocyte and tubular injury resulting from the mesangial cell inflammatory response [34, 35].

A phase 2, double-blinded, randomised control trial (clinical trial number NCT02942381) looked at the effects of Hydroxychloroquine on proteinuria in patients with IgA nephropathy. The trial concluded that Hydroxychloroquine when combined with optimised RAAS inhibition, proteinuria was significantly reduced in patients with IgA nephropathy over 6 months without evidence of adverse events [36]. Additionally, a retrospective case series analysed eight patients, treated with Hydroxychloroquine and RAAS inhibition, with a known diagnosis of X-linked AS and persistent haematuria and proteinuria. In seven patients, after one month of treatment, a reduction in both haematuria and proteinuria was recorded. This was sustained over a six-month period [37].

### Anti-MicroRNA-21

MicroRNAs are short non-coding RNAs that can regulate gene expression. MicroRNA-21 is widely expressed in multiple cell types in the kidney and is upregulated in some types of kidney disease. A single micro-RNA can silence the expression of several functionally related genes, thus meaning micro-RNA can function in an analogous but reciprocal way to transcription factors [38]. Increased levels of MicroRNA-21 are associated with increased gene expression modulating the initial tissue repair response following acute/chronic kidney injury, followed by inflammation and subsequent fibrosis in the tubulointerstitium in the kidney. In *Col4a3*^*−/−*^ Alport syndrome mice, the anti-fibrotic effects of anti-microRNA-21 were demonstrated, these protective effects were increased further when combined with ACE inhibitor therapy [39].

The HERA trial (clinical trial number NCT02855268) began in 2019 with the aim of assessing the effect of an anti-miRNA-21 drug (SAR339375) on reducing the rate of decline in kidney function in AS patients. The trial was later stopped in 2022 as the effect on kidney function had not been found. Additional trials, further assessing anti-MicroRNA-21 pharmaceuticals in the management of AS are expected to be underway in the near future.

### Genome editing therapy

This experimental technique aims to edit defective genes with the aim of curing a disease. It can be executed in various ways. A number of approaches have already been applied in AS studies specifically. Daga et al. isolated podocyte-lineage cells from urine of patients with AS. The study then demonstrated that in X-linked and autosomal dominant AS, the use of a self-inactivating dual-plasmid approach can result in double strand breaks caused by CRISPR/Cas9 which go on to be corrected through non-homologous end joining, thus generating deletions and insertions in specific defective genes. This method resulted in very high correction rates which ranged from 44% in the *COL4A3* gene to 58% in the *COL4A5* gene, both genes known to be defective in patients with AS [40]. The CRISPR/Cas9 gene therapy is thought to have huge potential in helping to restore a functional GBM, especially in the early stages of AS. However, it should be noted this is in its very early stages of development because at present this type of therapy has not yet been transitioned to in vivo experiments [40, 41]. It should also be highlighted that the benefits of novel treatments must always be weighed up against potential risks.

## Conclusion

Looking ahead, the combination of ongoing clinical trials, new pharmaceutical advancements and further research to help gain a greater understanding of disease mechanisms, the future of how we manage AS looks promising. The role of Finerenone in the management of AS needs to be explored further, alongside several additional drugs, such as SGLT2 inhibitors and gene editing therapeutics. There is extreme optimism for improved and better treatments for CKD associated with AS in the near future.

## Data Availability

Data sharing not applicable to this article as no datasets were generated or analysed during the current study.
